# Biomechanical Superiority of the Chinese Knotting Technique in Posterior Cruciate Ligament Reconstruction: A Cadaveric Study

**DOI:** 10.1111/os.70403

**Published:** 2026-08-07

**Authors:** Zhengliang Shi, Rui Han, Ping Yuan, Zhaohui Ruan, Hongmai Yang, Wenting Tang, Kaiquan Li, Xianguang Yang, Yang Yu, Yanlin Li

**Affiliations:** ^1^ Departments of Sports Medicine The First Affiliated Hospital of Kunming Medical University Kunming People's Republic of China; ^2^ International Medical Department The First Affiliated Hospital of Kunming Medical University Kunming People's Republic of China

**Keywords:** biomechanical properties, cadaveric specimens, internal tension‐relieving augmentation suture, posterior cruciate ligament

## Abstract

**Objective:**

We previously developed a novel internal tension‐relieving augmentation suture, the Chinese knotting technique (CKT), which demonstrated better knee kinematic recovery and clinical outcomes when assisting posterior cruciate ligament (PCL) reconstruction. This study evaluated the biomechanical effect of the CKT in cadaveric PCL reconstruction and provided experimental evidence for its clinical application.

**Methods:**

Ten fresh‐frozen adult knee joint specimens were randomly divided into the conventional reconstruction (CR; *n* = 5) and CKT (*n* = 5) groups. First, all native PCLs underwent cyclic fatigue testing with a standardized fixation method, and the relaxation length was recorded. The native PCL was then transected and reconstructed. After reconstruction, the same fixation method was applied for biomechanical testing to measure stiffness, elastic modulus, maximum load, and relaxation length.

**Results:**

No statistically significant differences were observed between the two groups in donor age (54.40 ± 5.94 years vs 56.60 ± 5.98 years), gender distribution (male: 80.00% vs. 60.00%), or specimen side (left: 60.00% vs. 40.00%) (*p* > 0.05). All specimens underwent successful reconstruction and testing with no instances of tissue damage or fixation abnormalities. The biomechanical testing revealed no statistically significant differences in stiffness (68.60 ± 8.62 N/mm vs. 65.40 ± 12.14 N/mm) or elastic modulus (147.60 ± 5.68 MPa vs. 142.60 ± 1.52 MPa) between the two groups (*p* > 0.05). However, the CKT group demonstrated a significantly higher maximum load (603.20 ± 77.44 N) compared with the CR group (442.20 ± 20.52 N) and a significantly lower relaxation length (1.50 ± 0.09 mm vs. 2.46 ± 0.02 mm) (*p* < 0.05).

**Conclusion:**

The CKT preserved the basic mechanical properties of the graft in PCL reconstruction. This technique significantly increased the maximum load and reduced residual deformation after cyclic loading, thereby providing enhanced biomechanical protection for the graft. This approach may offer technical support for early postoperative rehabilitation and a reduced risk of graft failure.

## Graphical abstract

In cadaveric PCL reconstruction, the Chinese knotting technique preserves graft mechanics, significantly increases maximum load, and limits elongation, supporting early rehabilitation and reducing failure risk.

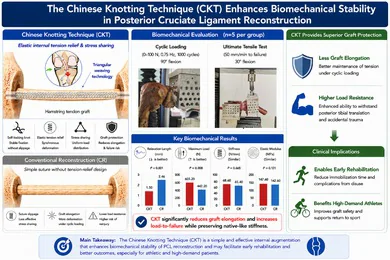

## 1 Introduction

Posterior cruciate ligament (PCL) is a key structure within a sophisticated biomechanical system, primarily responsible for maintaining posterior stability of the knee [1]. Epidemiological data indicate that PCL injuries comprise approximately 3% of all knee injuries in the general population and account for as many as 44% of all knee ligament injuries [2]. These injuries occur more frequently in men (77.54%) than in women (22.46%), with a majority of patients (60.96%) being younger than 35 years [3]. The most common mechanisms of injury are traffic accidents (approximately 45%) and sports‐related activities (approximately 40%) [4]. Because the PCL has a limited capacity for self‐healing, arthroscopic reconstruction is the standard of care for complete ruptures or clinically significant laxity. The success of this procedure directly influences the restoration of knee stability and the patient's functional recovery [5].

Current PCL reconstruction techniques primarily utilize autografts, allografts, or synthetic ligaments. Among these, autologous hamstring tendons (semitendinosus and gracilis) are frequently preferred due to their favorable biocompatibility and low rate of donor‐site morbidity [6]. A critical limitation of this approach, however, is the initially insufficient biomechanical strength of the graft. Following implantation, the autograft undergoes a well‐characterized “ligamentization” cascade comprising sequential phases of necrosis, revascularization, and collagen remodeling [7]. Importantly, rather than acquiring immediate mechanical competence, the graft paradoxically experiences a substantial decline in tensile properties during the initial inflammatory phase, typically reaching its weakest state at approximately 4–6 weeks postoperatively. This is followed by a slow, progressive recovery in mechanical strength as revascularization and organized collagen deposition advance, a process that generally extends over 6–12 months [8]. Consequently, this transient reduction in load‐bearing capacity, coupled with the mechanical demands of early mobilization, constitutes a primary driver of graft elongation, laxity, and ultimately, reconstruction failure. A further concern is that the intraoperative diameter of autologous hamstring tendon grafts is often less than 8 mm, with some studies reporting averages below 7 mm [9]. This diameter is frequently insufficient to match the anatomical dimensions of the native PCL, exacerbating the risk of uneven stress distribution and graft failure. Studies indicate that the failure rate after PCL reconstruction can reach 46.7%, representing a major challenge to successful surgical outcomes [10].

To optimize the graft's mechanical environment and mitigate early failure risk, various techniques have been investigated. Suture augmentation has been shown to improve anteroposterior stability following PCL reconstruction, restoring posterior tibial translation to near‐physiological levels [11, 12]. However, conventional suture augmentation often suffers from limited long‐term anti‐relaxation efficacy due to asynchronous deformation between the suture and graft under cyclic loading, as well as local suture cutting into the graft. As an emerging mechanical protection strategy, internal bracing—which involves placing a load‐sharing structure in or around the graft—offers a potential solution to balance the graft's vulnerability during ligamentization with the need for early rehabilitation. However, high‐strength internal brace devices, while providing robust initial fixation, may introduce a significant stress‐shielding effect. To address these limitations, we previously developed and clinically applied a novel internal tension‐relieving augmentation suture (Chinese knotting technique, CKT) for the reconstruction of anterior cruciate ligament (ACL) and PCL, which demonstrated favorable early clinical outcomes and functional recovery [13, 14]. Unlike rigid internal braces or loose suture augmentation, the CKT is designed to provide “elastic internal tension relief” (Figure 1). Under physiological loads, the graft remains the primary load‐bearing structure, ensuring adequate mechanical stimulation to support the ligamentization process, while the CKT synchronously deforms and shares stress without causing local cutting or stress shielding. This design aims to achieve a mechanical environment closer to that of the native PCL, offering early graft protection without compromising biological healing. However, despite these promising clinical results, a systematic biomechanical evaluation of this technique in a controlled, near‐physiological environment is still lacking. Specifically, there is a scarcity of quantitative data from cadaveric studies, which are considered the gold standard for such evaluations due to their accurate replication of human knee anatomy and biomechanics.

**FIGURE 1:**
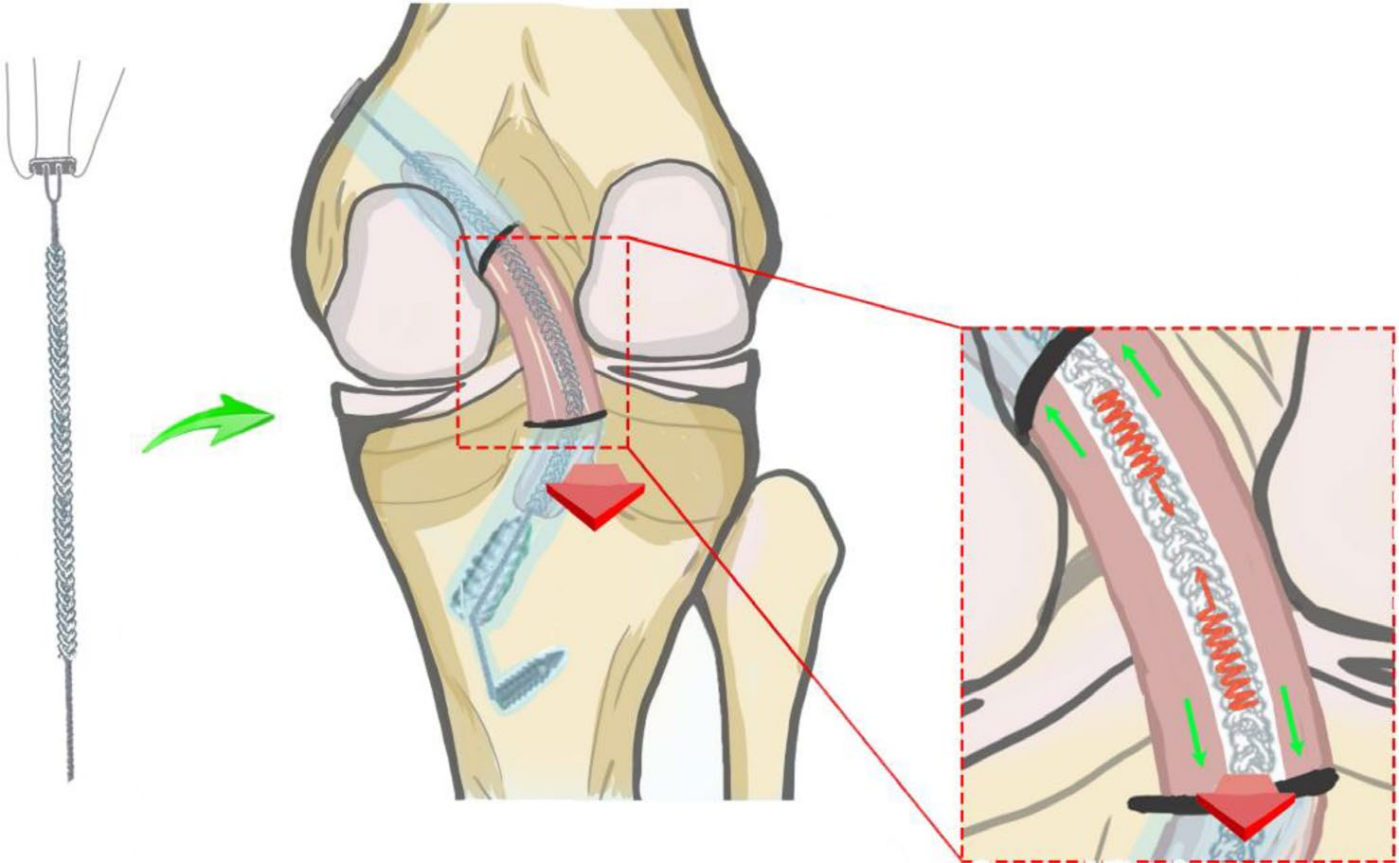
Macroscopic mechanical diagrams related to CKT.

Therefore, this study utilized human cadaveric knee specimens to investigate the biomechanical properties of the CKT for PCL reconstruction with hamstring tendon grafts. We assessed the effect of this augmentation on key graft parameters, including maximum load, elastic modulus, and elongation, through biomechanical testing [15]. This study aims to provide experimental evidence for the biomechanical efficacy of this technique and to elucidate the mechanism of its “elastic internal tension relief” [16]. The findings are expected to offer a theoretical foundation and technical reference for optimizing PCL reconstruction protocols and reducing postoperative graft failure, thereby contributing to more precise and effective management of PCL injuries.

## 2 Materials and Methods

### 2.1 Study Subjects

#### 2.1.1 Source of Specimens

Ten fresh‐frozen adult knee joint specimens were procured from self‐donated bodies of the Department of Anatomy, Southern Medical University. All donors had no documented history of knee joint trauma, surgery, or degenerative disease, thus meeting the study inclusion criteria. This study strictly adheres to the Declaration of Helsinki and has been approved by the Ethics Committee of the First Affiliated Hospital of Kunming Medical University (2024‐L‐280).

#### 2.1.2 Specimen Pretreatment

Following retrieval, specimens were thawed gradually in a 4°C refrigerator for approximately 24 h until they reached room temperature. This protocol was implemented to prevent alterations in tissue mechanical properties due to repeated freeze–thaw cycles [17]. Each specimen was secured on a multi‐functional anatomical table. A layered dissection was then performed, centered on the knee joint space, using the following procedure:

First, the skin and subcutaneous fat were dissected bluntly using hemostats. The muscles of the quadriceps femoris and hamstrings were then stripped layer by layer along their fiber orientation to expose the ipsilateral semitendinosus and gracilis tendons (Figure 2). The semitendinosus and gracilis tendons were isolated, and any adherent muscle fibers and fascia were removed. The two prepared tendons were folded in half and were sutured together to create a composite graft composed of four tendon strands. Each composite tendon was trimmed into a graft measuring 24–26 cm in length and 8–9 mm in diameter. Both ends of the grafts were reinforced by weaving with a #2 ultra‐high molecular weight polyethylene (UHMWPE) suture (Figure 3). The prepared grafts were labeled and stored in a sealed container with 0.9% normal saline for subsequent use in PCL reconstruction.

**FIGURE 2:**
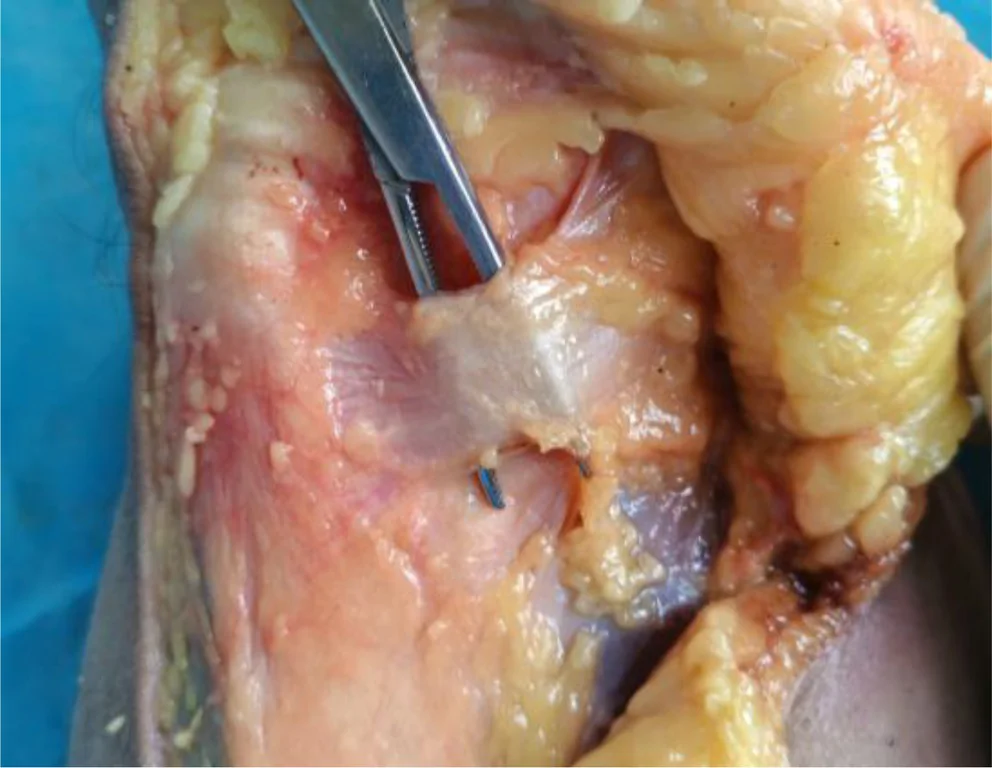
Exposure of the ipsilateral semitendinosus and gracilis tendons in the specimen.

**FIGURE 3:**
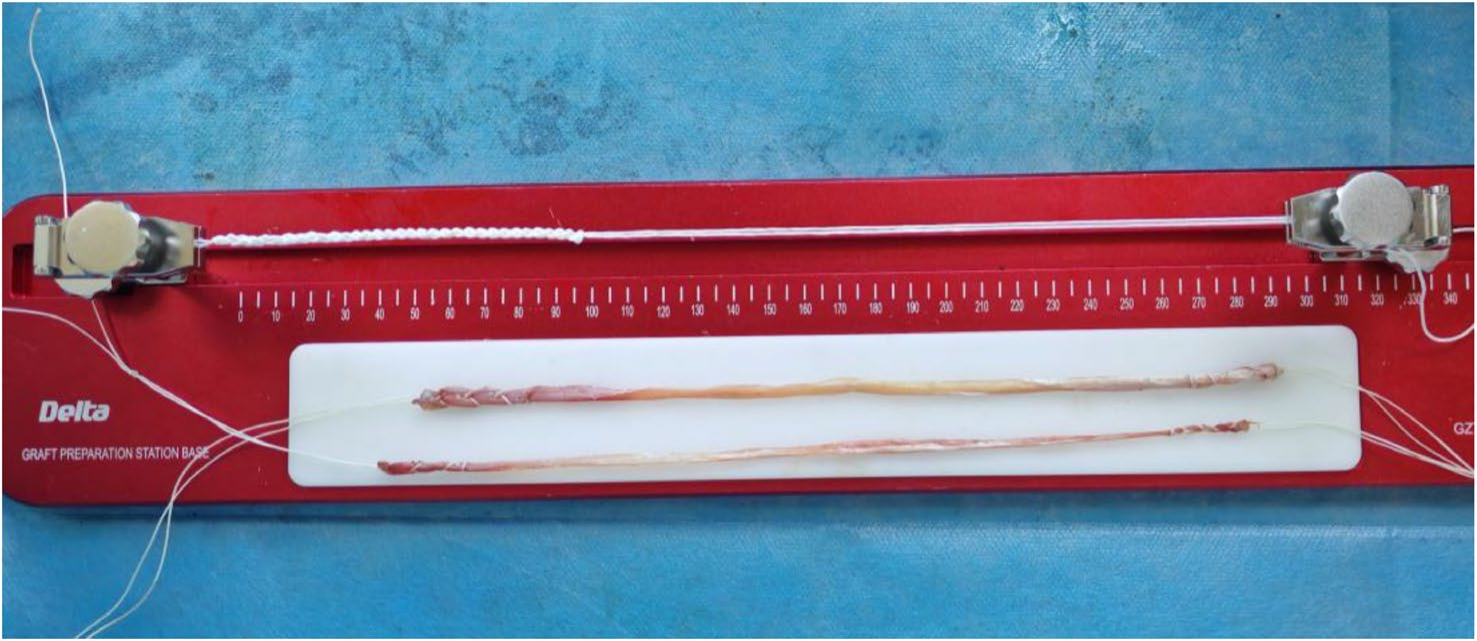
Pretreatment of the semitendinosus and gracilis tendons.

The fascia and synovial tissue surrounding the joint capsule were carefully excised to fully expose the key anatomical structures, including the medial collateral ligament, lateral collateral ligament, ACL, menisci, and PCL.

With the PCL identified as the primary structure to be preserved, non‐target tissues were selectively dissected. Specifically, the medial and lateral collateral ligaments were sharply transected at their insertion sites. Throughout the dissection, the PCL fiber bundles were gently lifted with forceps to verify the integrity of its femoral insertion on the medial condyle and its tibial insertion in the intercondylar fossa. This ensured that no fiber rupture or avulsion fractures occurred at the insertion sites. All procedures were conducted with the tissues continually moistened with normal saline to prevent desiccation and preserve tissue viability [18].

#### 2.1.3 Cyclic Testing of the Native PCL

Biomechanical cyclic testing was first conducted on the native PCL of all specimens to quantify its relaxation properties. Specimens were secured using a custom PCL biomechanical testing fixture, as detailed below.

*Tibial fixation*: To prevent bone splitting, pre‐drilling was performed at preset points on the proximal tibia (located > 5 cm from the tibial plateau and aligned parallel to the coronal plane) using a drill bit 0.5 mm smaller in diameter than the fixation rods. Two cylindrical rods were then inserted through the tibial drill holes and the corresponding frame holes to achieve rigid fixation. These rods were positioned parallel and symmetrical, with a 15 mm spacing, to prevent axial movement or lateral displacement.

*Femoral fixation*: The supracondylar portion of the femur was potted and secured in a wedge‐shaped fixture compatible with the testing machine. The fixture's inner wall featured anti‐slip ridges to enhance grip, ensuring the femoral long axis was aligned with the loading axis of the testing machine.

*Angle adjustment*: The knee joint was flexed to 90° to simulate the clinical posterior drawer test position. This angle was locked with a positioning pin, maintaining a flexion angle fluctuation of ≤ 1° throughout testing.

Following fixation, cyclic testing was performed using a BOSE material testing system (ELF‐3510AT, Bose Inc., USA). A sinusoidal tensile load ranging from 0 to 100 N was applied at a frequency of 0.75 Hz for 1000 cycles. The testing environment was maintained at 25°C and 60% relative humidity. The system recorded load–displacement data in real‐time at a sampling frequency of 500 Hz. The relaxation length of the native PCL was recorded upon completion of the cyclic testing. Subsequently, the native PCL was sharply transected at its femoral and tibial insertion sites. Care was taken to ensure complete ligament transection while preserving the bone structure in areas designated for subsequent tunnel placement. All specimens, along with the prepared grafts, were then returned to sealed containers with 0.9% normal saline for storage until the subsequent grouping and reconstruction procedures.

### 2.2 Experimental Grouping and Reconstruction Method

#### 2.2.1 Grouping Protocol

Ten knee joint specimens were randomly assigned to one of two groups (*n* = 5 per group) using a random number table.

The conventional reconstruction (CR) group underwent standard anatomical PCL reconstruction with a #2 UHMWPE suture.

The CKT group underwent anatomical PCL reconstruction augmented with a #2 UHMWPE suture and a novel internal tension‐relieving suture.

#### 2.2.2 Graft Preparation

Graft preparation differed between the groups. In the CR group, pretreated autologous hamstring tendons (semitendinosus and gracilis tendons) were used directly as grafts.

In the CKT group, the pretreated grafts were modified with an internal tension‐relieving suture, prepared as follows [14] (Figure 4). First, the tension‐relieving suture was passed through its own self‐locking loop to form a knot with one‐way stress self‐locking properties. This design effectively limits slippage under load, ensuring stable fixation within the loop. The pretreated graft tendon was then passed through this loop, which was positioned at the graft's center. Finally, the tension‐relieving suture was wrapped and fixed longitudinally along the graft's middle segment. Both ends of the suture were aligned with the graft's woven suture, securing the tension‐relieving suture along the central axis of the graft without loosening or displacement.

**FIGURE 4:**
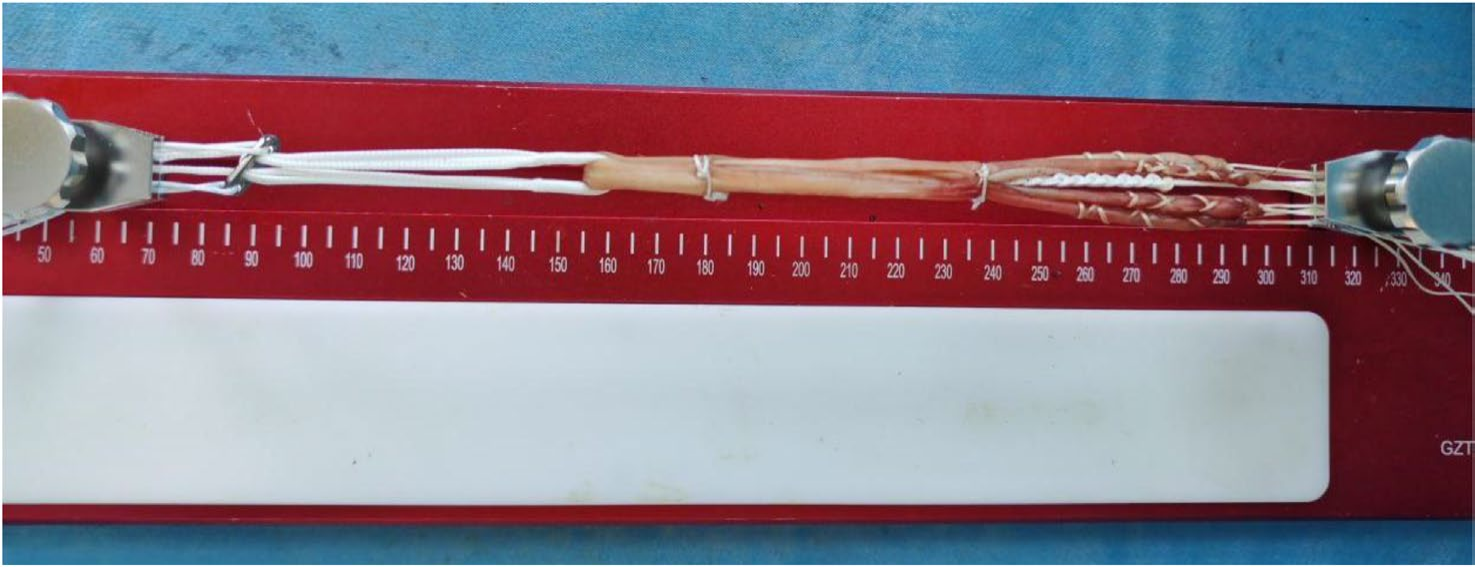
The CKT suture located at the center of the graft tendon.

#### 2.2.3 PCL Reconstruction

All reconstruction procedures were performed by the same team of senior sports medicine physicians. The specific steps were as follows:

*Bone tunnel creation*: Centered on the PCL's anatomical insertion site, femoral and tibial bone tunnels were created using a cannulated drill with a diameter matching the graft (8–9 mm). The femoral tunnel was positioned at the center of the PCL footprint on the medial femoral condyle. The tibial tunnel was located in the posterolateral PCL insertion area of the tibial intercondylar fossa.

*Graft implantation and fixation*: In the CR group, the prepared graft was passed through the bone tunnel from the femur to the tibia. The femoral end was fixed with an adjustable loop plate (Beijing Deyidamei Co. Ltd., China). The tibial end was fixed using an interference screw (Beijing Deyidamei Co. Ltd., China) combined with an external row fixation anchor (Beijing Deyidamei Co. Ltd., China). Before final fixation, the knee joint was cycled through flexion and extension to confirm no impingement [19] (Figure 5). In the CKT group, the suture‐augmented graft was implanted identically. During fixation, both ends of the tension‐relieving suture were tensioned synchronously with the graft. The tension was adjusted to a level that did not cause obvious tensile deformation of the graft. All other fixation procedures were identical to the CR group (Figure 6).

**FIGURE 5:**
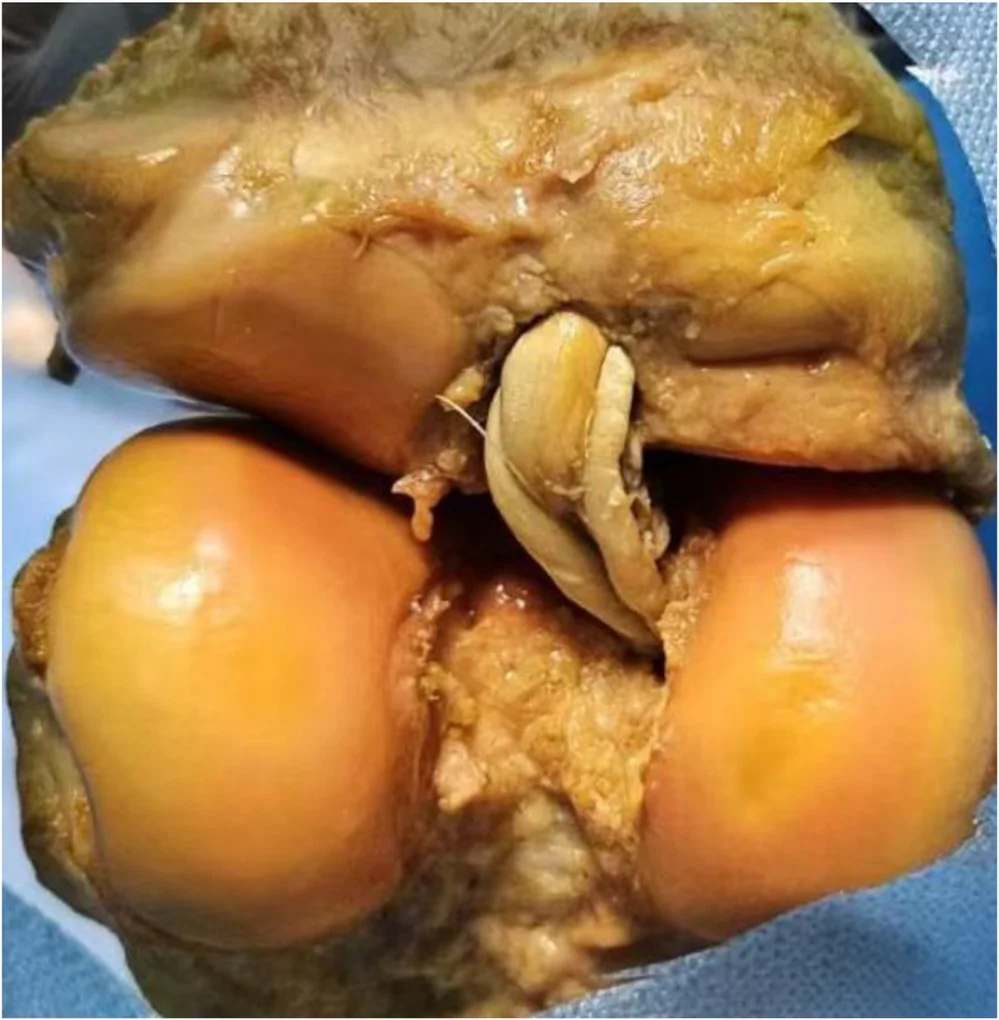
Femur‐tibia‐graft tendon complex after reconstruction in the CR group.

**FIGURE 6:**
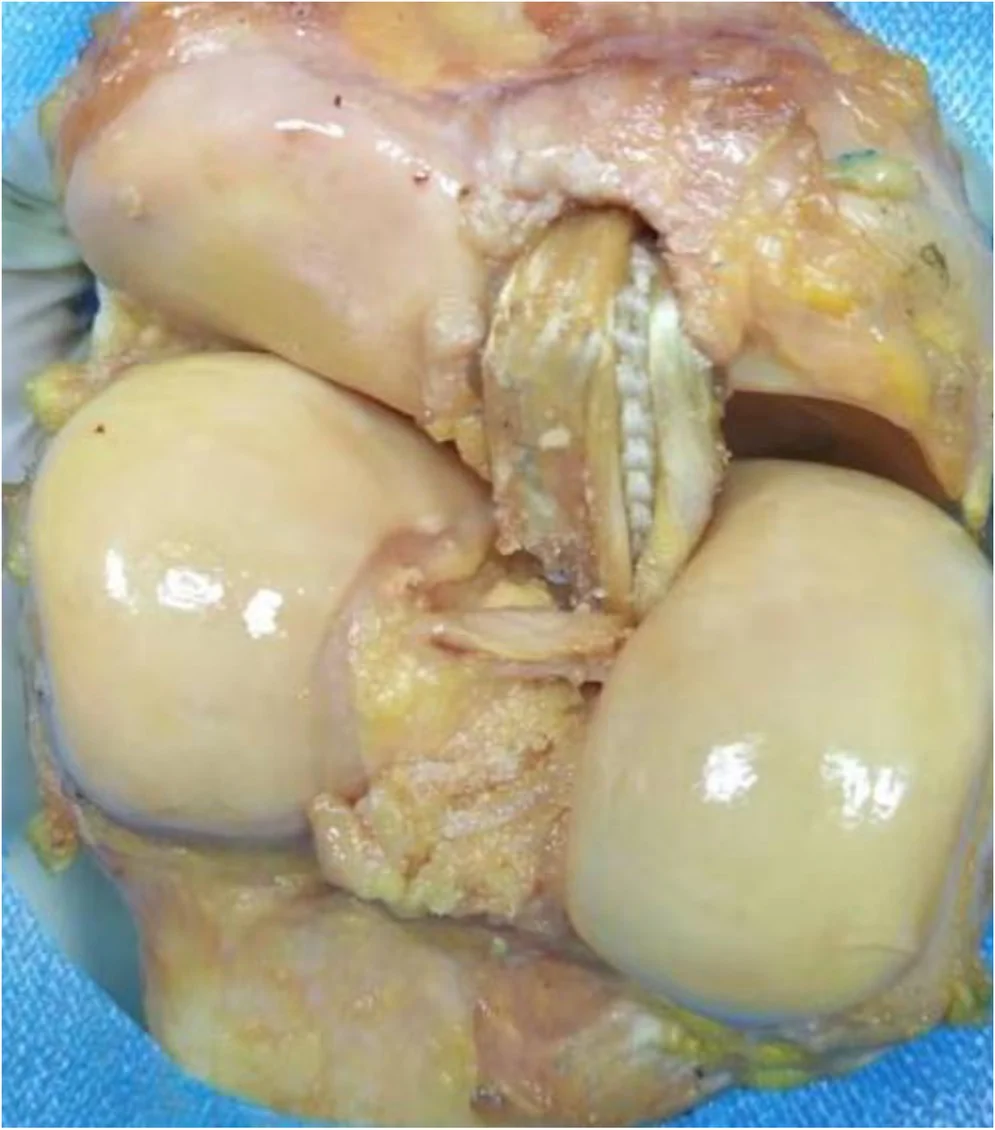
Femur‐tibia‐graft tendon complex after reconstruction in the CKT group.

*Postoperative processing and preparation for biomechanical testing*: Following reconstruction, the graft position and fixation stability were verified. Excess tissue around the bone tunnels was removed to isolate the femur‐graft‐tibia complex. This complex was then mounted on a PCL biomechanical testing fixture using the same method described for the native PCL. This involved rigid fixation of the tibial end with two cylindrical fixtures and embedded fixation of the femoral end with a wedge‐shaped bone fixture, with the knee joint locked at 90° of flexion. The complex was stored in saline‐soaked sealed containers and underwent biomechanical testing within 2 h.

### 2.3 Biomechanical Testing

#### 2.3.1 Specimen Fixation (Consistency With Native PCL Testing)

The fixation method for cyclic fatigue testing was consistent with that used for the native PCL. For the subsequent tensile testing, the knee joint was flexed to 30°, replicating the reverse Lachman test position. This angle was secured with a positioning pin to prevent deviation during testing. The transition from cyclic to tensile testing proceeded as follows: Following cyclic testing on the BOSE material testing system (ELF‐3510AT, Bose Inc., USA), the specimen was transferred to an electronic universal testing machine (Model: ATES6010, Guangzhou Aojin Industry Co. Ltd., China). The distal femur was secured to the upper clamp with a custom fixture, and the proximal tibia was fixed to the lower clamp. Throughout fixation, the natural anatomical alignment between the tibial and femoral axes in flexion was preserved to avoid tibial rotation or lateral displacement.

#### 2.3.2 Specimen Preloading

All tests were conducted in a controlled environment of 25°C and 60% relative humidity using an ElectroForce biomechanical testing system (Bose Corporation, USA). Prior to formal testing, all specimens underwent a preconditioning protocol to mitigate the effects of tissue relaxation and creep. This involved applying a constant 80 N load at a constant rate for 5 min [20]. Once the load–displacement curve stabilized, the system's load and displacement readings were zeroed to return to the initial testing state.

#### 2.3.3 Biomechanical Measurement and Recording

Biomechanical testing commenced immediately after preconditioning [21]. The protocol consisted of two consecutive phases:

Cyclic Fatigue Testing: A sinusoidal load ranging from 0 to 100 N was applied at a frequency of 0.75 Hz for 1000 cycles [18, 22]. The system recorded load–displacement data in real‐time at a sampling frequency of 500 Hz. Upon completion, the graft's relaxation length was documented for comparison with the native PCL (Figure 7).

**FIGURE 7:**
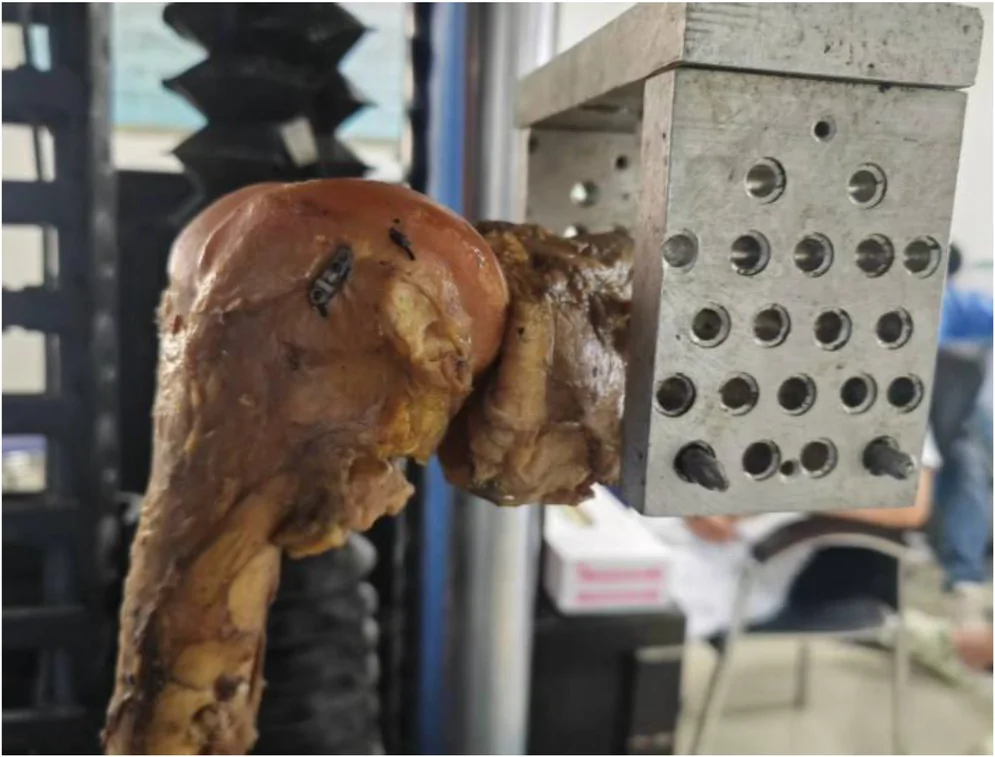
Cyclic fatigue testing with fixation at the 90° position.

Ultimate Tensile Testing: Immediately following cyclic testing, the upper clamp was displaced from the lower clamp at a rate of 50 mm/min. This motion simulated posterior tibial translation relative to the femur in the reverse Lachman test [23]. Testing continued until mechanical failure, defined as a sudden load drop of ≥ 80% from the peak value or visible graft rupture. Load–displacement data were continuously recorded at 500 Hz, and the ultimate failure load (the peak load attained during tension) was captured (Figure 8).

**FIGURE 8:**
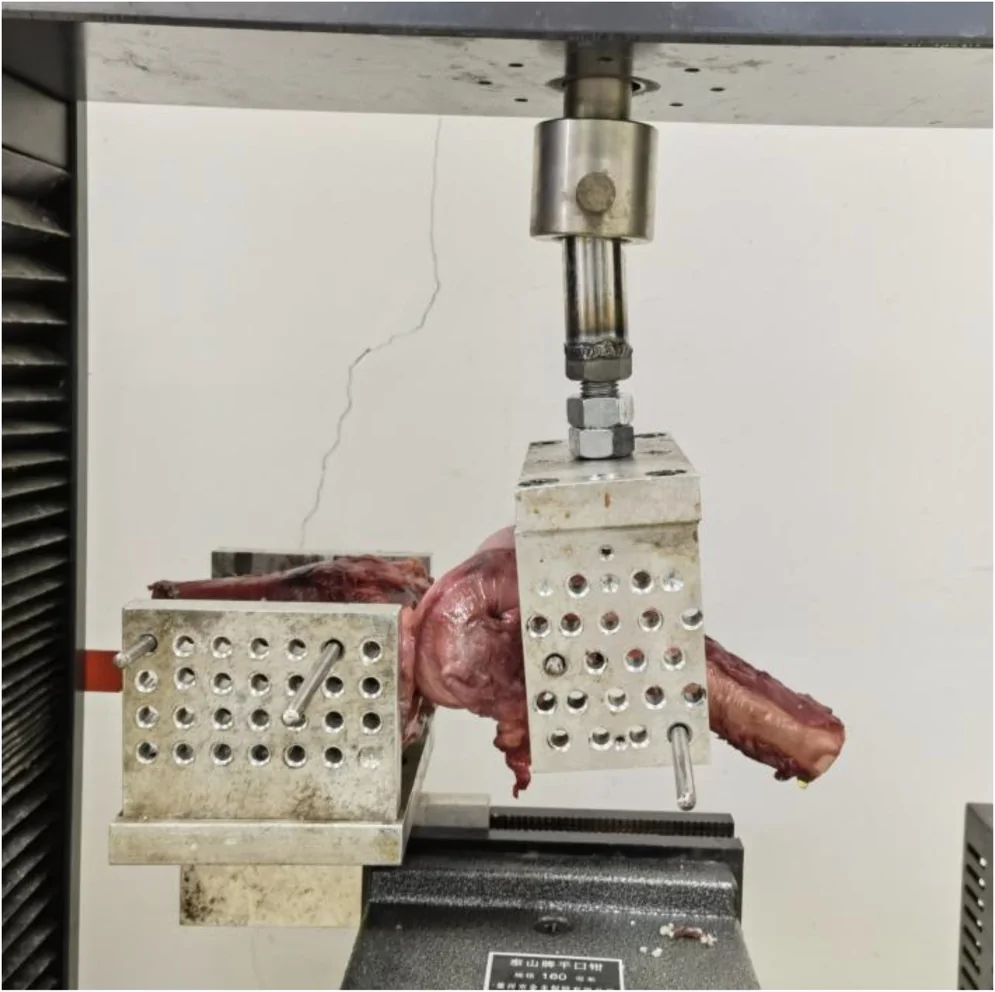
Axial tensile testing simulating the reverse Lachman test.

### 2.4 Biomechanical Outcome Measures

Four primary biomechanical parameters were evaluated. All data were acquired via the testing system's data acquisition module at a sampling frequency of 500 Hz and verified anatomically. The definitions and calculation methods for each parameter are detailed below.

#### 2.4.1 Maximum Load (Unit: N)

Defined as the peak tensile load sustained by the native PCL prior to mechanical failure, which was determined by a sudden load drop of ≥ 80% from the peak or upon visual confirmation of graft rupture. Tensile testing was performed at a rate of 50 mm/min. The maximum load was automatically identified by the testing system from the load–displacement curve.

#### 2.4.2 Relaxation Length (Unit: mm)

This measure quantifies the residual deformation of the specimen following cyclic loading. It was defined as the gauge length recorded after cyclic fatigue testing (0.75 Hz, 0–100 N, 1000 cycles). The gauge points were demarcated at the effective ends of the graft, spanning from the femoral fixation edge to the tibial fixation edge. Length measurements were recorded in real time by the testing system's displacement sensor with an accuracy of 0.01 mm.

#### 2.4.3 Stiffness (Unit: N/mm)

Stiffness, reflecting a structure's resistance to elastic deformation, was calculated from the linear elastic region of the load–displacement curve obtained during the tensile phase of cyclic testing. The value was derived as the slope of the curve, representing the change in load per unit change in displacement [24].

#### 2.4.4 Elastic Modulus (Unit: MPa)

The elastic modulus, which represents the intrinsic property of a material to resist elastic deformation, was calculated from the linear elastic region of the stress–strain curve derived from the tensile phase of cyclic testing [25].

### 2.5 Statistical Analysis

All statistical analyses were performed using IBM SPSS Statistics software (Version 26.0). Continuous data are presented as mean ± standard deviation (SD). The normality of all continuous data distributions was confirmed using the Shapiro–Wilk test (*p* > 0.05), and homogeneity of variance was verified with Levene's test, thereby satisfying the prerequisites for parametric tests. Categorical data (e.g., donor sex, knee side) are expressed as frequencies (*n*). Intergroup comparisons of continuous data (including donor age and biomechanical outcome measures) were conducted using unpaired, two‐tailed Student's *t*‐tests. The Pearson chi‐square test was employed to assess the distribution of categorical data (donor sex, specimen side) between groups. A two‐sided *p*‐value of < 0.05 was considered statistically significant for all tests.

## 3 Results

### 3.1 PCL Reconstruction of Specimens

Primary anatomical PCL reconstruction was successfully performed on all 10 knee specimens. The procedure was completed without adverse events, such as graft tear, bone tunnel fracture, or avulsion at the PCL insertion sites. In the CR group (*n* = 5), the graft was implanted and fixed according to the standard protocol. In the CKT group (*n* = 5), all procedures, including the creation of a self‐locking knot with the novel augmentation suture, graft loop positioning, and suture fixation, were accomplished successfully. No instances of suture loosening, displacement, or deviation of the graft loop from its central axis were observed.

Following reconstruction, visual inspection and manual traction testing confirmed proper graft positioning at the native PCL insertion site in all specimens. No excessive folding or abnormal tension was noted within the bone tunnels. During simulated knee flexion and extension, the graft demonstrated an absence of impingement within the intercondylar fossa and maintained appropriate tension, while the knee joint retained normal anatomical alignment.

Baseline characteristics, including donor sex, specimen side, and age, were comparable between the two groups, with no statistically significant differences (all *p* > 0.05; Table 1).

**TABLE 1:** Baseline information analysis.

Project	Distribution	Group (%)		Total	*χ* ^2^	*p*
		CKT	CR			
Gender	Male	4 (80.00)	3 (60.00)	7 (70.00)	0.476	0.490
	Female	1 (20.00)	2 (40.00)	3 (30.00)		
Specimen side	Left	3 (60.00)	2 (40.00)	5 (50.00)	0.400	0.527
	Right	2 (40.00)	3 (60.00)	5 (50.00)		
Age (years)		54.40 ± 5.94	56.60 ± 5.98	−0.583	−0.583	0.576

### 3.2 Biomechanical Test Results of PCL Reconstruction in the Two Groups

All 10 specimens successfully underwent cyclic loading and ultimate tensile testing. The testing protocol was completed without complications, such as fixture loosening or premature graft failure, ensuring complete data collection. The results are summarized in Figure 9 and Table 2.

**FIGURE 9:**
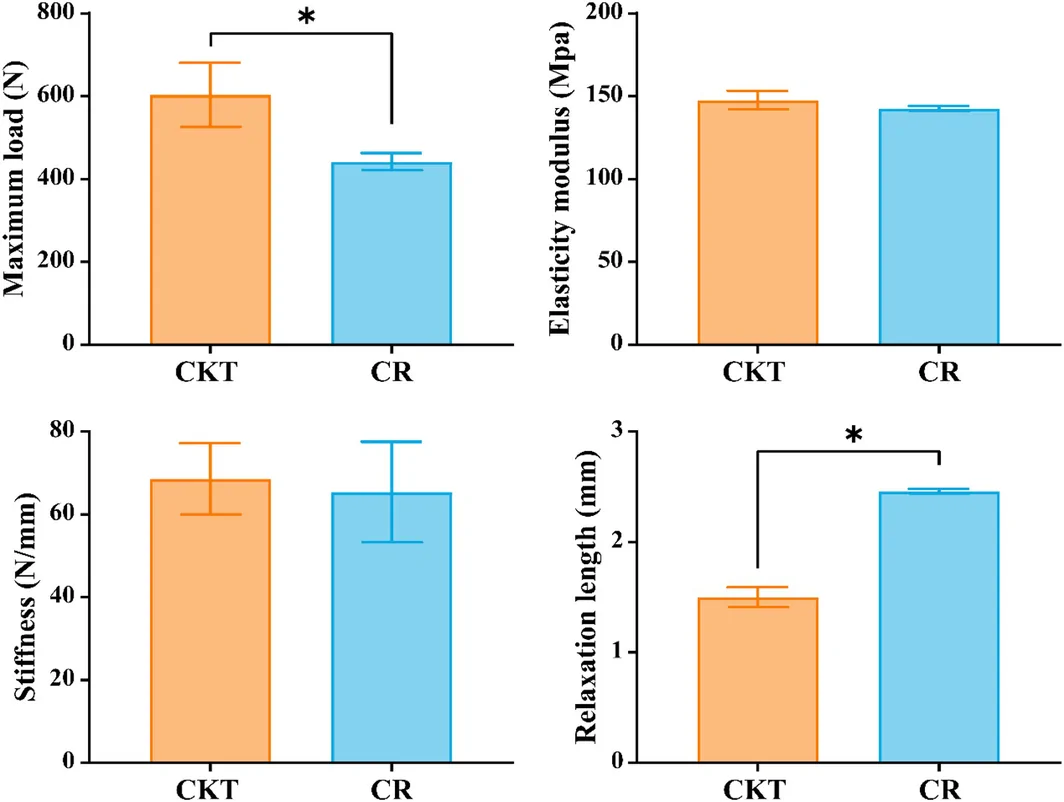
Histogram of biomechanical test results.

**TABLE 2:** Intra‐articular biomechanical testing regarding elastic modulus, maximum load and relaxation length.

Group	Stiffness (N/mm)	Elasticity modulus (MPa)	Maximum load (N)	Relaxation length (mm)
CKT (*n* = 5)	68.60 ± 8.62	147.60 ± 5.68	603.20 ± 77.44	1.50 ± 0.09
CR (*n* = 5)	65.40 ± 12.14	142.60 ± 1.52	442.20 ± 20.52	2.46 ± 0.02
*t*	0.450	1.901	4.494	−24.687
*p*	0.665	0.121	0.008*	0.000*

*Note:* Compared with CR group, *p < 0.05.

#### 3.2.1 Stiffness and Elastic Modulus

No significant differences in material properties were observed between the two groups. The stiffness was 68.40 ± 8.68 N/mm for the CKT group and 65.40 ± 12.14 N/mm for the CR group (*p* > 0.05). Similarly, the elastic modulus was 147.60 ± 5.68 MPa and 142.60 ± 1.52 MPa for the two groups, respectively (*p* > 0.05).

#### 3.2.2 Maximum Load

The CKT group demonstrated a significantly greater maximum load (603.20 ± 77.44 N) compared to the CR group (442.20 ± 20.52 N), and this difference was statistically significant (*p* < 0.05).

#### 3.2.3 Relaxation Length

The internal tension‐relieving technique resulted in significantly less graft elongation, with a relaxation length of 1.50 ± 0.09 mm, which was significantly shorter than the 2.46 ± 0.02 mm observed in the CR group (*p* < 0.05; Figure 10).

**FIGURE 10:**
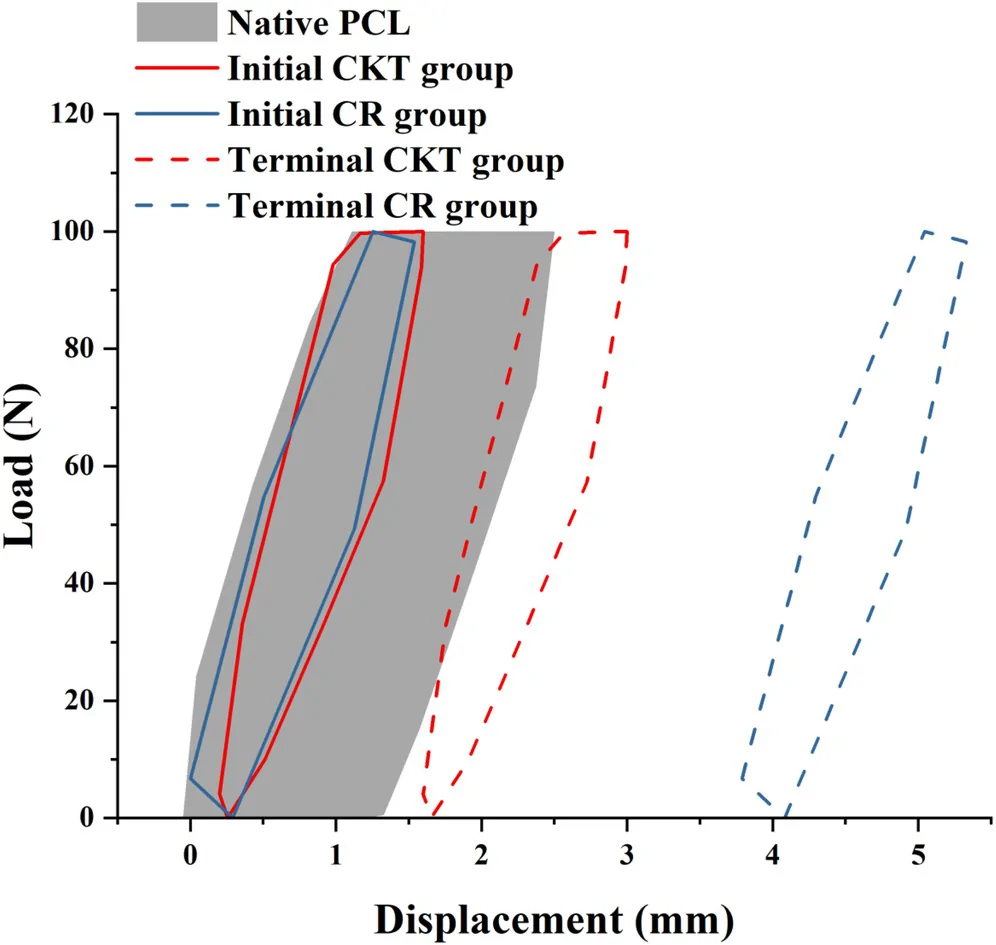
Load–displacement curves of ligament cyclic fatigue testing. The gray shaded area denotes the relaxation range of the native posterior cruciate ligament (PCL). Load–displacement curves for the grafts are shown for the initial (solid lines) and final (dashed lines) stages of cyclic fatigue testing, performed with a 0.75 Hz sinusoidal frequency under a load range of 0–100 N for 1000 cycles. The CKT group and the CR group are represented by red and blue lines, respectively. Analysis of the curves reveals that the final cyclic response of the CKT group more closely approximates the native PCL's relaxation range. Furthermore, this group exhibited less graft elongation following cyclic testing, indicating that the CKT effectively mitigates residual graft deformation after cyclic loading.

## 4 Discussion

### 4.1 Core Value of Biomechanical Verification for the CKT

#### 4.1.1 Protective Effect of the CKT on PCL Grafts

Biomechanical verification is a critical prerequisite for the clinical translation of novel medical technologies, as it clarifies core efficacy and mitigates application risks. This study evaluated the CKT for PCL reconstruction, which was designed with the primary goal of providing early postoperative graft protection. Its core function is “elastic internal tension relief,” achieved through a specialized “triangular weaving technology” [13, 14]. This design imparts mechanical compatibility to the suture, enabling synchronous deformation and uniform stress‐sharing with the graft under load, thereby preventing local stress concentrations.

We quantitatively evaluated this design using fresh‐frozen human cadaveric knees. Cyclic loading (0.75 Hz, 0–100 N) simulated daily activities, while tensile testing (50 mm/min) simulated accidental posterior tibial translation. The CKT group demonstrated significantly less graft relaxation (1.50 ± 0.09 mm) than the CR group (2.46 ± 0.02 mm; *p* < 0.05). This indicates that the elastic tension‐relief function effectively reduces residual graft deformation after cyclic loading, thereby mitigating graft elongation. Furthermore, the maximum load was significantly higher in the CKT group (603.20 ± 77.44 N vs. 442.20 ± 20.52 N; *p* < 0.05).

It is important to note that the maximum load in both groups remained below the reported ultimate load of the native PCL [22]. This finding aligns with the established understanding in the field of ligament reconstruction, as grafts—whether traditional or augmented—inherently differ from the native ligament in their anatomical structure and collagen fiber arrangement [26, 27]. The core value of our findings, however, lies in the significant improvement in maximum load provided by the CKT. This load exceeds that of typical ordinary hamstring tendon grafts used clinically, confirming that the CKT enhances graft failure resistance through stress‐sharing, thereby offering more robust mechanical support for early postoperative protection.

#### 4.1.2 Potential of the CKT in Inhibiting Posterior Tibial Translation

The primary functional goal of PCL reconstruction is to restore posterior knee stability by inhibiting excessive posterior tibial translation [28, 29]. Our results suggest the CKT contributes to this goal through a dual‐mechanism of action. The significantly reduced graft relaxation (*p* < 0.05) indicates superior maintenance of graft morphology and tension during cyclic loading, which simulates rehabilitation exercises. This helps prevent a decrease in posterior constraint capacity caused by graft elongation. Concurrently, the higher maximum load (*p* < 0.05) provides enhanced resistance to accidental postoperative trauma. Together, these factors create a complementary “dynamic protection and static constraint” mechanism that indirectly reduces posterior tibial translation.

This inference is consistent with other biomechanical studies demonstrating that internal augmentation can significantly reduce posterior knee laxity [12, 30]. The 0.96 mm reduction in relaxation length observed in our study further suggests the potential of this technique to inhibit posterior tibial translation, providing preliminary biomechanical data to support its clinical value in restoring posterior knee joint stability.

### 4.2 Rationality of the Biomechanical Design of the Cadaver Model and Advantages of the Control Design in This Study

This study utilized fresh‐frozen human cadaveric knees and employed a parallel control design (*n* = 5 per group). The baseline comparability between groups was confirmed for donor age, sex, and knee joint status (all *p* > 0.05). To ensure procedural standardization, a single senior surgeon performed all PCL reconstructions using identical bone tunnel positioning and graft fixation techniques (interference screw on the femoral end; adjustable loop plate on the tibial end). This approach minimizes that observed differences are attributable to the surgical technique rather than procedural variability.

A custom biomechanical fixture was used to maintain a physiological loading posture, closely replicating the postoperative stress environment of the knee. The testing protocol was designed to integrate clinical examination standards and actual injury mechanisms, ensuring high clinical relevance.

For cyclic loading, which simulates daily activities, the knee was fixed at 90° of flexion. This is the standard position for the clinical posterior drawer test and a common angle for PCL stress during activities like squatting or climbing stairs [31]. Applying a load of 0–100 N at 0.75 Hz in this position realistically reproduces the dynamic stretching forces on the graft during early rehabilitation.

For ultimate tensile testing, which simulates failure from a traumatic event, a knee flexion of 30° was used. This angle corresponds to the reverse Lachman test and places the PCL under physiological tension [32, 33], mirroring the mechanism of injury from a posterior force on the flexed knee. A tensile rate of 50 mm/min was applied until failure to accurately assess the graft's load‐to‐failure resistance in a clinically relevant scenario.

The selected outcome measures—stiffness, elastic modulus, maximum load, and relaxation length—form a comprehensive mechanical profile, addressing key clinical concerns after PCL reconstruction:

*Maximum load*: This metric directly reflects the graft's ability to resist failure under high stress, a primary cause of early postoperative complications [34]. The significantly higher maximum load in the CKT group (*p* < 0.05) suggests an improved capacity to withstand posterior trauma, thereby reducing the risk of early rupture.

*Relaxation length*: This indicator corresponds to early postoperative stability. Graft elongation is a key factor in joint instability and delayed rehabilitation. The significantly lower relaxation in the CKT group (*p* < 0.05), along with its load–displacement curve being closer to the native PCL's range, indicates superior maintenance of graft tension during cyclic loading. This provides a safer mechanical environment for initiating early rehabilitation exercises, such as straight leg raises and partial weight‐bearing.

*Stiffness and elastic modulus*: The absence of significant differences between groups for these properties (both *p* > 0.05) confirms that the CKT provides “auxiliary stress sharing” without altering the fundamental mechanical properties of the graft. This is a critical finding, as it aligns with the clinical goal of enhancing stability without restricting the knee's range of motion.

### 4.3 Mechanistic Interpretation of the Dissociated Biomechanical Parameters

A central finding of our study is the dissociation between material‐level and structural‐level parameters: the CKT significantly improved maximum load and relaxation resistance while leaving stiffness and elastic modulus unchanged. This divergence is not paradoxical but mechanistically instructive.

Stiffness and elastic modulus are intrinsic graft properties, determined by collagen architecture, cross‐linking, and tissue hydration—factors unaltered by the addition of an augmentation suture. The CKT is tensioned without pre‐stressing the resting graft; the suture does not engage within the linear elastic range, and the measured slope of the load–displacement curve remains graft‐dominant. This preserves physiological joint laxity during low‐demand activities, avoiding the “over‐constraint” that accelerates adjacent cartilage degeneration.

Maximum load and relaxation length, by contrast, are structural composite parameters. Under cyclic loading, the self‐locking knot engages progressively as the graft enters its nonlinear toe region. The longitudinal suture shares cyclic tensile stress, reducing peak strain amplitude and mitigating interfibrillar creep—directly reflected in the 0.96 mm reduction in relaxation length. In the ultimate tensile phase, the suture provides a secondary load‐bearing pathway that maintains integrity after initial fiber failure, raising the failure threshold by approximately 160 N.

The knot configuration also modifies the mode of failure. In the CR group, rupture uniformly originated at the femoral tunnel aperture—the well‐documented “killer turn” where graft bending concentrates stress. In CKT specimens, the longitudinal wrapping offloaded this focal stress toward the intra‐articular midsubstance. Moreover, the intact suture often preserved partial continuity after graft rupture, offering structural redundancy. This mechanical protection is particularly valuable during the 6‐ to 12‐week ligamentization window, when the graft undergoes maximal biological weakening and remains most vulnerable to elongation or failure.

In essence, the CKT achieves “iso‐elastic reinforcement”: preservation of elastic behavior under physiological loads, combined with enhanced resistance to failure under high or repetitive stress. This property profile aligns with the clinical goal of enabling early rehabilitation without compromising long‐term graft integrity.

### 4.4 Clinical Translation Significance of the CKT

#### 4.4.1 Potential Value in Promoting Early Postoperative Rehabilitation of PCL Reconstruction

A central challenge in postoperative rehabilitation after PCL reconstruction is balancing the “need for early joint stability” against the “risk of complications from prolonged immobilization.” Conventional protocols often restrict posterior tibial stress for over 3 months to protect the graft, which can lead to quadriceps atrophy (reported incidence of 28.9%) and joint stiffness [35].

Our findings demonstrate that the internal tension‐relieving technique results in a graft with significantly less relaxation (*p* < 0.05) and a higher maximum load (*p* < 0.05), while maintaining mechanical compatibility with the native graft, as evidenced by the similar stiffness and elastic modulus between groups (both *p* > 0.05). These properties suggest that excessive activity restriction may be unnecessary in the early postoperative phase. The augmentation suture shares loads during rehabilitation, protecting the graft from excessive elongation, while its synchronous deformation characteristic helps prevent graft disuse atrophy by avoiding stress‐shielding [36]. This profile aligns with the modern sports medicine principle of “function first, early rehabilitation, and timely return to sport” [37]. Consequently, this technique may shorten the required period of strict immobilization and reduce the incidence of associated rehabilitation complications.

#### 4.4.2 Adapting to PCL Reconstruction Needs of Populations With High Sports Demands

Young, athletic individuals (aged 18–35) represent a high‐risk group for PCL injuries and have a strong desire to return to sport. However, the insufficient load tolerance of grafts after traditional reconstruction contributes to a low sports return rate of only 44% [38]. The daily and sporting activities of this population (e.g., jogging, jumping) place high mechanical demands on the PCL graft, requiring robust strength to withstand dynamic loads [39].

In this study, the maximum load of the graft in the CKT group reached 603.20 ± 77.44 N, significantly exceeding the 442.20 ± 20.52 N observed in the CR group (*p* < 0.05). This mechanical advantage better equips the graft to withstand the forces encountered in athletic scenarios, thereby reducing the risk of failure due to overloading. Furthermore, the secure, non‐slipping fixation structure of the CKT ensures consistent central stress‐sharing even under high‐frequency, high‐load conditions. This design adapts to the functional demands of an athletic knee, offering a technical solution for improving return‐to‐sport rates in this demanding patient population.

### 4.5 Limitations of the Study

The sample size (*n* = 5 per group) was determined by a priori power calculation based on our pilot data, which provided > 80% power to detect the observed difference in maximum load (~160 N, pooled SD ~56 N, *α* = 0.05). The significant between‐group differences in both maximum load and relaxation length (*p* < 0.01 and *p* < 0.001) confirm adequate sensitivity for our primary endpoints. Nonetheless, the modest sample precludes subgroup analyses and may limit the generalizability of absolute secondary parameters due to donor variability. As a time‐zero cadaveric model, this study does not replicate in vivo biological remodeling or synovial effects. Thus, clinical translation requires further validation through animal or prospective human studies with larger cohorts. Second, as a cadaveric investigation, we could not account for the biological ligamentization process, which influences graft mechanical properties over time. Our results therefore reflect time‐zero biomechanical behavior and require validation through animal or clinical studies. Third, the cyclic loading (0–100 N, 0.75 Hz, 1000 cycles) was selected to mimic early‐stage rehabilitation loads, not high‐intensity activities. Future fatigue testing under higher amplitudes would better assess long‐term durability. Fourth, our protocol applied only uniaxial tension at fixed flexion angles, missing rotational and shear forces that occur in vivo. More sophisticated multi‐axial testing is needed.

## 5 Conclusion

This cadaveric study confirms that the CKT provides effective biomechanical reinforcement for hamstring tendon grafts in posterior cruciate ligament reconstruction without compromising the intrinsic elastic behavior of the graft. The preservation of stiffness and elastic modulus indicates that the augmentation suture does not induce rigid stress shielding, while the significant gains in maximum load and resistance to cyclic relaxation demonstrate a genuine load‐sharing capacity that engages predominantly under higher or repetitive stress. From a clinical perspective, these properties support a shift from prolonged, restrictive immobilization toward earlier, safer rehabilitation, as the augmented graft maintains tension and structural integrity during the vulnerable 6‐ to 12‐week ligamentization window when biological weakening is most pronounced.

## Author Contributions

**Zhengliang Shi:** conceptualization, methodology, data curation, investigation, validation, formal analysis, funding acquisition, visualization, project administration, writing – original draft. **Rui Han:** conceptualization, methodology, investigation, validation, formal analysis, visualization, writing – review and editing. **Wenting Tang:** conceptualization, methodology, investigation, validation, resources, writing – review and editing. **Yang Yu:** conceptualization, investigation, validation, writing – review and editing. **Yanlin Li:** conceptualization, data curation, funding acquisition, project administration, resources, writing – review and editing. **Zhaohui Ruan:** conceptualization, methodology, investigation, validation, resources, writing – review and editing, formal analysis. **Xianguang Yang:** conceptualization, methodology, investigation, validation, writing – review and editing. **Ping Yuan:** conceptualization, methodology, investigation, validation, formal analysis, visualization, writing – review and editing. **Kaiquan Li:** conceptualization, methodology, investigation, validation, writing – review and editing. **Hongmai Yang:** conceptualization, methodology, investigation, validation, formal analysis, writing – review and editing.

## Funding

This study was supported by the Yunnan Provincial Academician (Expert) Workstation (No. 202405AF140051), the Scientific Research Project of the General Administration of Sport of China, 2025 (No. 18), the Yunnan Provincial Social Development Special Project (No. 202403AC100025), and the Yunnan Provincial International Joint Innovation Platform (No. 202403AP140023).

## Ethics Statement

Fresh‐frozen adult knee joint specimens were obtained from self‐donated bodies. This study strictly adheres to the Declaration of Helsinki and has been approved by the Ethics Committee of the First Affiliated Hospital of Kunming Medical University (2024‐L‐280).

## Conflicts of Interest

The authors declare no conflicts of interest.

## Data Availability

The data that support the findings of this study are available from the corresponding author upon reasonable request.
